# Combined treatment for a large primary cardiac sarcoma: a case report

**DOI:** 10.1186/s40792-019-0658-x

**Published:** 2019-06-17

**Authors:** Shinji Akishima, Akito Imai, Yoshiharu Enomoto, Osamu Shigeta

**Affiliations:** Department of Cardiovascular Surgery, Ibaraki Prefectural Central Hospital, 6528 Koibuchi, Kasama-shi, Ibaraki, 309-1793 Japan

**Keywords:** Undifferentiated pleomorphic sarcoma, Primary cardiac tumor, Acute heart failure, Proton beam radiotherapy, Molecular targeted drugs, Combined therapy

## Abstract

**Background:**

Undifferentiated pleomorphic sarcoma (UPS) as a primary cardiac tumor is rare, with extremely poor prognosis owing to high recurrence and invasion. We encountered a patient who presented with a primary cardiac tumor incarcerating the mitral valve and who was in a shock state.

**Case presentation:**

A 41-year-old man was transported emergently to our hospital owing to acute respiratory distress and hemoptysis. He was diagnosed with acute left heart failure caused by a large mass in the left atrium (LA) that obstructed cardiac blood flow, as revealed by imaging study findings, and he underwent an emergency open-heart surgery for tumor resection. He was pathologically diagnosed with UPS invading the muscle layer of the LA. However, after receiving combined therapy for local recurrence and distant metastasis, including proton beam radiotherapy and chemotherapy with molecularly targeted drugs, he could return to work for 2 more years after surgery.

**Conclusion:**

In this study, we reported the case of a patient who was in a state of shock state owing to the presence of UPS in the LA. The patient underwent an emergency surgery and received combined therapy. He survived for 2 more years after an initial diagnosis, without active local recurrence and distant metastasis.

## Background

In this study, we report the case of a patient who was in a shock state owing to the presence of a large undifferentiated pleomorphic sarcoma (UPS) in the left atrium (LA). The patient underwent an emergency surgery and received combined therapy. He survived and returned to work for 2 more years after surgery despite local recurrence and distant metastasis.

## Case presentation

**Fig. 1:**
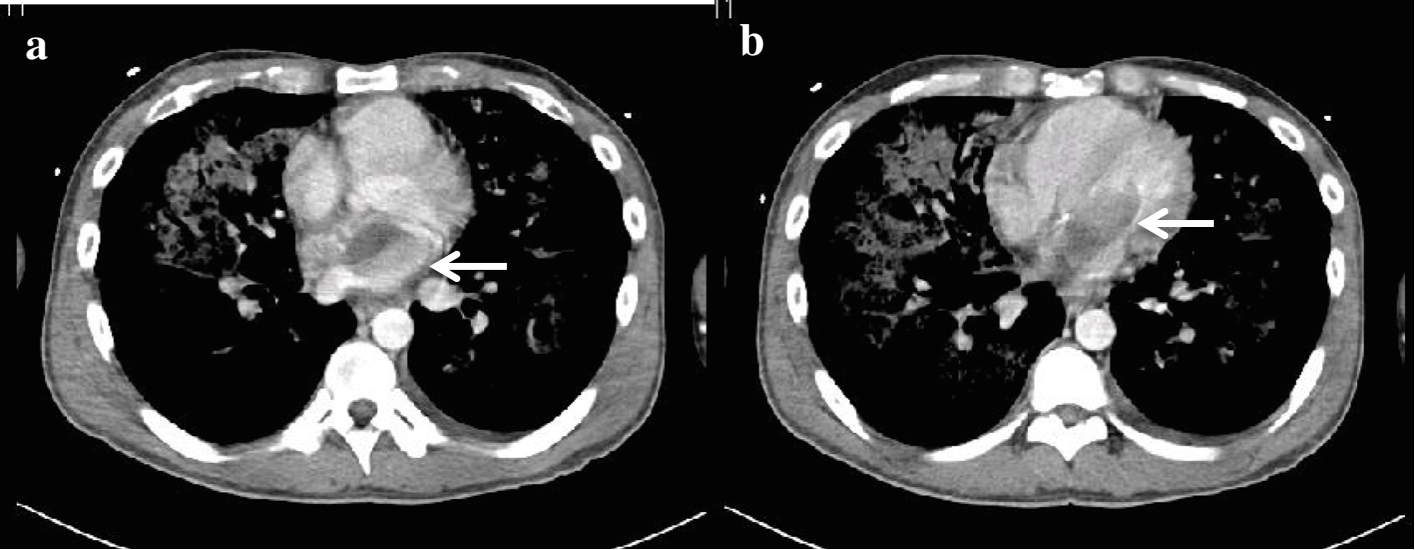
Preoperative enhanced computed tomography scan. It revealed a large tumor in the left atrium (arrow) (**a**), incarcerating into the mitral annulus (arrow) (**b**)

A 41-year-old man suffering from the bilateral knee and ankle arthralgia for several months was transported emergently to our hospital owing to acute respiratory distress and hemoptysis. Upon arrival, he was in a shock state. Chest roentgenography revealed severe pulmonary congestion; cardiac echogram revealed a large mass in the LA that incarcerated into the mitral valve. Additionally, chest computed tomography (CT) revealed a tumor in the LA; thus, he was diagnosed with acute left heart failure caused by the mass that obstructed cardiac blood flow (Fig. 1).

**Fig. 2:**
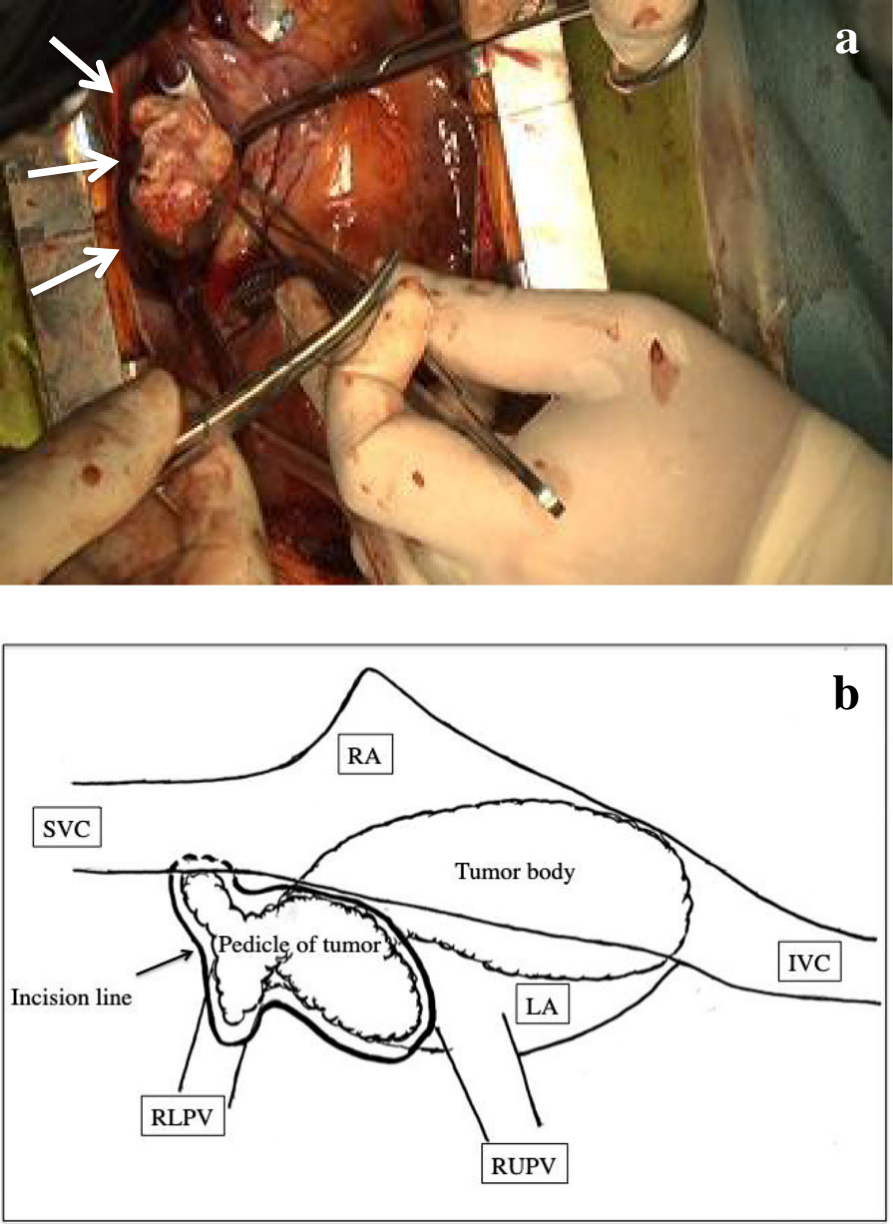
Intraoperative view. A tumor was extirpated through an incision in the right side of the LA wall (white arrows) (**a**). A schema of the existence of the tumor in the LA. The tumor pedicle was widely and irregularly attached to the LA wall, which extended to the right upper pulmonary vein, and the tumor body was bound for mitral annulus (**b**)

An emergency surgery was performed under cardiac arrest with extracorporeal circulation, which was established in the usual manner with bicaval direct cannulation. Because of the dimensions of the tumor and its pedicle attachment, we could approach through both the wall incisions on the right-side LA from the right upper pulmonary vein and atrioseptostomy from the right atrium. The tumor pedicle widely and irregularly originated from the right upper and posterior LA wall and extended to the lateral LA wall, which included the right upper pulmonary vein. The tumor was visibly extirpated and invaded the LA wall (Fig. 2). The shape and function of the mitral valve were intact, and the large defect in the LA wall was reconstructed using a bovine pericardial patch. It was 159 min under extracorporeal circulation, and the aortic cross-clamping time was 123 min.

**Fig. 3:**
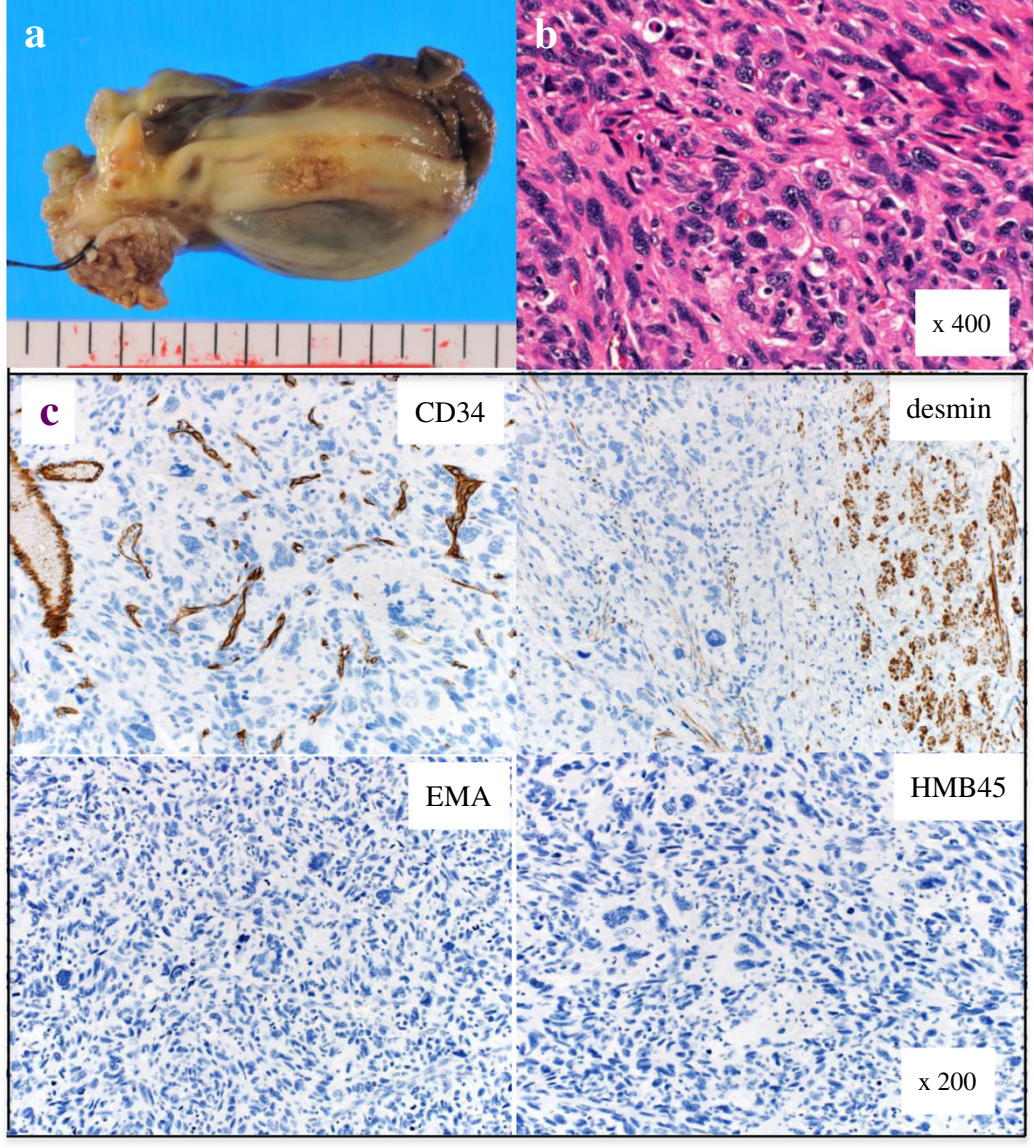
Excised cardiac tumor. **a** The tumor occupied the left atrium cavity (75 × 37 × 30 mm). **b** Pathological findings of the tumor showed undifferentiated high-grade pleomorphic sarcoma with an irregular spindle or multi-nucleated giant cells (hematoxylin and eosin staining). **c** Immunohistochemical staining. Tumor cells were negative for CD34, desmin, EMA, and HMB45

Extracorporeal circulation weaning and post-operative course were uneventful, and arthralgia in both lower limbs disappeared immediately after surgery. The pathological diagnosis was UPS with clear resection margins (R0 resection), which invaded the atrial muscular layer (Fig. 3). Subsequently, as imaging studies soon and 3 months after surgery did not reveal tumor presence, we decided to adopt a more suitable treatment strategy without involving adjuvant therapy after surgery if UPS relapse or metastasis occurred. Specifically, we planned to perform re-surgical resection or proton radiotherapy for recurred or metastatic tumors. In addition, we planned to initiate systemic chemotherapy using a target organ drug or other anti-malignant tumor agents for distant metastasis depending on the local and general conditions of the patient. He was discharged 20 days after surgery without additional treatment and was able to work 2 months after discharge.

**Fig. 4:**
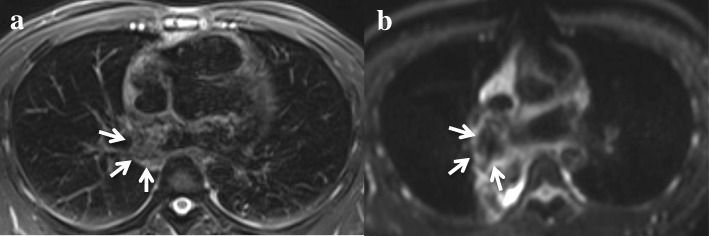
Local recurrence on magnetic resonance imaging (MRI). **a** At 7 months after surgery, before proton beam radiotherapy, recurrent tumor shadow was detected (arrows). **b** After proton beam radiotherapy, the tumor shadow was hardly detected (arrows)

However, local recurrence in the LA was observed on positron emission CT (PET) and other imaging studies 7 months after surgery (Fig. 4a). A tumor was detected on the posterior LA wall adjacent to the incision line of previous surgery. He again experienced arthralgia in both lower limbs. Thus, we selected radiotherapy with proton beam as treatment, and a dose of 75 Gy was delivered to the recurrent tumor in 30 fractions for 45 days. No tumor was observed in the LA on imaging performed 2 months after radiotherapy as an outpatient (Fig. 4b).

**Fig. 5:**
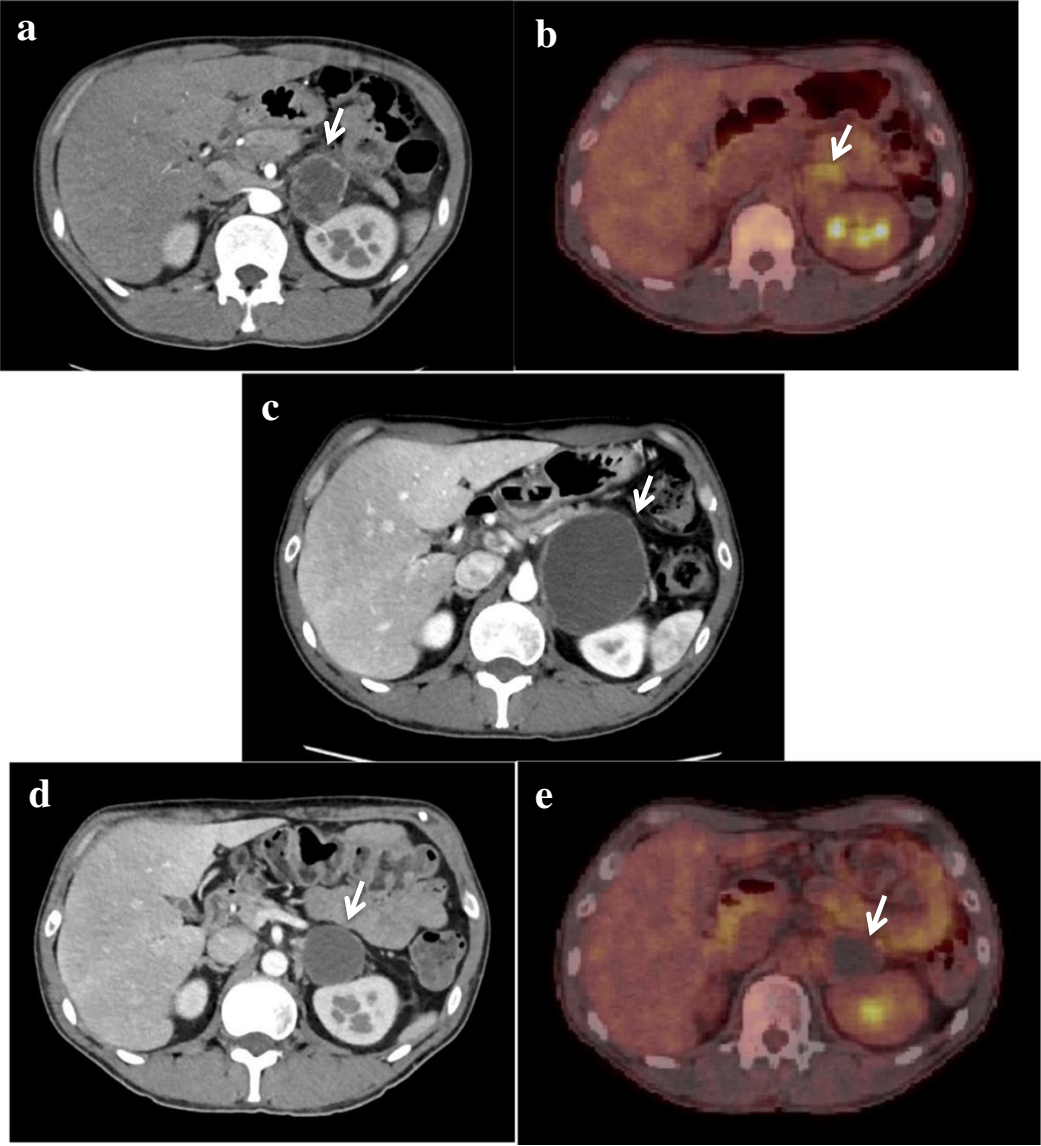
Distant metastasis to the left adrenal gland on imaging studies. **a** CT before the second radiotherapy with proton beam. **b** PET before the second radiotherapy with proton beam; remarkable fluorodeoxyglucose (FDG) uptake was found. **c** CT at the end of the second radiotherapy; larger but cystic and non-enhanced mass was observed. **d**, **e** CT and PET 8 months after the completion of the second radiotherapy; the size of the mass was remarkably reduced, and the blood flow nor the FDG uptake was not observed

After 6 months, the second local recurrence at a different site in the LA and distant metastasis to the left adrenal gland were simultaneously observed on the results of several imaging tests. In the left adrenal gland, a large solid tumor with an irregular surface and abundant blood flow was observed on enhanced CT scan (Fig. 5a), and remarkable fluorodeoxyglucose (FDG) uptake was found on positron emission tomography/CT scan (Fig. 5b). Proton beam radiation for both tumors was selected to conserve the left kidney function. A dose of 60 Gy was delivered to the tumor in 30 fractions in the LA and 46 Gy in 23 fractions in the left adrenal gland. Moreover, chemotherapy with pazopanib hydrochloride (800 mg/day), a tyrosine-kinase inhibitor (molecularly targeted drug), was used in combination radiotherapy. At the end of the second radiotherapy, a larger but cystic and non-enhanced mass in the left adrenal gland was observed on CT (Fig. 5c). While the patient was receiving chemotherapy for 8 months after the completion of the second radiotherapy, the size of the left adrenal mass apparently reduced. Moreover, neither blood flow nor fluorodeoxyglucose (FDG) uptake of lesions in both the LA and left adrenal gland were revealed on positron emission CT scan (Fig. 5d, e).

Twenty-seven months after surgery, no active tumor was noted on any imaging result, and he returned to work without symptoms, including arthralgia of the lower limbs.

## Discussion

UPS as a primary cardiac tumor is extremely rare and accounts for approximately 1.7% of all cardiac tumors [1, 2]. The prognosis of UPS is poor owing to its highly aggressive and locally invasive characteristics. Mean patient survival period is approximately 1 year after diagnosis [2, 3]. Complete resection is the most recommended treatment for UPS [2–4], but is rarely accomplished in emergency cases because of the patients’ unstable condition and complex anatomical location of tumors upon initial examination.

In our case, the patient presented with acute and severe left heart failure and underwent emergency surgery. However, local recurrence occurred twice in the LA, and distant metastasis to the left adrenal gland was observed. Next, he was treated with proton beam radiation for the recurred tumors, and oral chemotherapy with tyrosine-kinase inhibitor (pazopanib), which is the only molecularly targeted drug authorized for treatment for sarcoma in our country, was additionally selected to suppress tumor activity [5, 6]. While he was receiving chemotherapy with pazopanib for 8 months after the completion of the second radiotherapy with proton beam, local recurrence and distant metastasis were not observed on CT and other imaging modalities.

An effective treatment for UPS except for surgical resection has not been thus far reported. Proton beam radiation is described as available for malignant tumors, including sarcoma [3]. However, there are restrictions for target sites and limitation of exposure radiation dose in those methods. Therefore, combined therapy, comprising radiotherapy, chemotherapy, and surgery may be effective for controlling UPS without majorly affecting the general condition of patients or causing damage to the organs and tissues around the targets.

Incidentally, our patient was suffering from arthralgia of both lower limbs several months before the initial diagnosis, and local recurrence and metastasis were observed; however, his pain disappeared after surgery and radiotherapy. There were no specific tumor markers on the blood test that indicated an activity of UPS. Thus, arthralgia may be a significant indicator of the tumor activity on clinical scenes.

## Conclusions

A patient with a primary cardiac tumor of UPS, which caused acute heart failure, was rescued by an emergency open-heart surgery. After receiving combined therapy for local recurrence and distant metastasis, including proton beam radiotherapy and chemotherapy with molecularly targeted drugs, he could return to work. Then, he survived for 2 more years after surgery under control of UPS.

## Data Availability

Please contact the author for data requests.

## Abbreviations

CT: Computed tomography
FDG: Fluorodeoxyglucose
LA: Left atrium
MRI: Magnetic resonance imaging
PET: Positron emission CT
UPS: Undifferentiated pleomorphic sarcoma
